# Development of Immunological Assays Based on *Leishmania donovani* Antigen for Diagnosis of Canine Visceral Leishmaniasis and Their Multicenter Evaluation in Brazil and Italy

**DOI:** 10.3389/fcimb.2022.914477

**Published:** 2022-07-01

**Authors:** Sarfaraz Ahmad Ejazi, Samiran Saha, Anirban Bhattacharyya, Sonali Das, Nathália Lopes Fontoura Mateus, Manoel Sebastião da Costa Lima, Herintha Coeto Neitzke-Abreu, Ivete Lopes de Mendonca, Carlos Henrique Nery Costa, Otoni Alves de Oliveira Melo, Marcia Almeida de Melo, Bartira Rossi-Bergmann, Raffaele Corso, Laura Manna, Nahid Ali

**Affiliations:** ^1^ CSIR-Indian Institute of Chemical Biology, Kolkata, India; ^2^ Faculty of Veterinary Medicine and Animal Science, Universidade Federal de Mato Grosso do Sul (UFMS), Campo Grande, Brazil; ^3^ Aggeu Magalhães Institute, Oswaldo Cruz Foundation, Recife, Pernambuco, Brazil; ^4^ Faculty of Health Sciences, Universidade Federal da Grande Dourados (UFGD), Mato Grosso do Sul, Brazil; ^5^ Departamento de Clínica e Cirurgia Veterinária, Universidade Federal do Piaui (UFPI), Teresina, Brazil; ^6^ Instituto de Biofísica Carlos Chagas Filho, Universidade Federal do Rio de Janeiro (UFRJ), Rio de Janeiro, Brazil; ^7^ Programa de Pós-Graduação em Ciência e Saude Animal, Universidade Federal de Campina Grande (UFCG), Campina Grande, Brazil; ^8^ Prevention Department, Distretto Sanitario 12, Unità Operativa di Prevenzione Collettiva, Azienda Sanitaria Locale Caserta, Caserta, Italy; ^9^ Department of Veterinary Medicine and Animal Production, University of Naples, Federico II, Naples, Italy

**Keywords:** *Leishmania*, serology, immunochromatographyic test (ICT), diagnosis, canine

## Abstract

Canine visceral leishmaniasis (CVL) due to *Leishmania infantum* infection is a zoonotic disease prevalent in the areas of South America and the Mediterranean. Infected dogs as reservoirs can contribute to disease transmission and can be a scourge to public health. Therefore, early diagnosis of infected dogs may play a pivotal role in circumscribing disease progression. Invasive tissue aspiration and insufficient serological methods impair a single assay for prompt CVL diagnosis. In the present study, we aimed to evaluate the potential of *Leishmania donovani* isolated membrane protein, LAg, for the diagnosis of CVL through immunological assays. Initially, enzyme-linked immunosorbent assay was done with Brazilian dog sera to evaluate the performance of LAg in diagnosing CVL and found sensitivity and specificity of 92.50% and 95%, respectively. The study further confirmed the diagnostic efficacy of LAg in a dipstick format. The dipstick test of canine sera from three centers in Brazil and one center in Italy collectively showed sensitivity values in the range of 53.33% to 100% in recognizing symptomatic dogs and specificity values between 75% and 100% to rule out healthy dogs. Moreover, a rapid immunochromatographic test was developed and optimized using LAg. This test was able to identify 94.73% of CVL of Brazilian origin with specificity of 97.29%. The current results highlight the reactive potential of the *L. donovani* antigen, LAg, for *L. infantum* CVL diagnosis and support our previous findings, which suggest the utility of LAg for the diagnosis of both *L. donovani* and *L. infantum* human VL in a variety of endemic regions. LAg as a diagnostic candidate may be employed to identify comprehensive CVL cases in epidemiological areas.

## Introduction

Visceral leishmaniasis (VL) is a severe illness in humans caused by the protozoan parasite *Leishmania donovani*, which is prevalent in South East Asia and North Africa and *Leishmania infantum*, which affects the population of South America and Mediterranean countries (Guerra et al., 2019; Toepp et al., 2019). The disease caused by *L. infantum* is zoonotic, in which both humans and animals can be infected by the parasite (Willen et al., 2019). Domestic dogs are more susceptible to infection that also expresses parasites in their skin, thus serving as the major reservoir for the parasite in addition to contributing to human transmission (Ribeiro et al., 2018). Canine VL (CVL) manifestations are highly variable and may or may not have clinical signs eventually during chronic infection (Ramirez et al., 2019). Infected dogs might have long-lasting symptoms ranging from self-limiting to severe ailments that lead to death (Maia and Campino, 2018). An array of clinical pathology is presented by the infected dogs including cutaneous lesions, lymphadenomegaly, muscular atrophy, onychogryphosis, and other common evidence such as fever, diarrhea, vomiting, and polyuria (Abbehusen et al., 2017). With respect to the clinical signs, infected dogs with confirmed CVL cases are classified as asymptomatic, oligosymptomatic, and symptomatic. Asymptomatic dogs are those who test positive without showing any symptoms. CVL-positive dogs with three clinical signs are suggestive of oligosymptomatic, whereas more than three signs are referred to as symptomatic (Contreras, 2018). Because of non-specific pathology and a wide spectrum of symptoms, a diagnostic approach based on a single test is not sufficient to detect CVL. In clinical settings, parasites are detected conventionally in tissue aspirates or biopsy samples through microscopy or *ex vivo* culture (Garcia et al., 2017). However, these methods are invasive and vary in sensitivity. Molecular assays such as the detection of parasitic DNA are circumscribed to equipped laboratories only (Oliveira-da-Silva et al., 2020). Clinically affected dogs with VL are often associated with inadequate Th1 responses, which lead to exacerbated production of non-protective immunoglobulins (Manna et al., 2008; Ribeiro et al., 2019). This fact encouraged the development of numerous serological methods for laboratory diagnosis of CVL. Indirect immunological methods of detecting serum antibodies such as enzyme-linked immunosorbent assay (ELISA) and indirect immunofluorescence assay are easier and more sensitive than parasitological method especially for symptomatic CVL. Hence, they were recommended by the Brazilian Ministry of Health until 2011 (Fraga et al., 2014). Despite their advantages, serological tests are not 100% sensitive and specific and are limited in their ability to detect early infection as well as to diagnose asymptomatic and oligosymptomatic dogs.

The immunochromatographic test (ICT) as a robust and handy method of diagnosis, owing to its ability to visually display the result and its applicability in resource-poor settings. The employment of ICTs in the onsite diagnosis of CVL, for example, lateral flow assay (LFA), has increased the diagnostic capacity in rural areas. The performance of ICT depends directly on the antigenicity of the leishmanial protein used. Several recombinant antigens such as *L. infantum* rK39, rK9, rK26, and rK28 have been investigated in the LFA format, either alone or in the dual-path platform (DPP®) (Contreras, 2018). As the prevalence of asymptomatic dogs is associated with a high risk of human transmission, a single diagnostic tool is still needed to screen a large number of infected dogs.

In the current study, we isolated highly reactive native membrane antigens, LAg, from promastigotes of *L. donovani* strain AG83 and evaluated its diagnostic potential against human canine VL. Further, we optimized LAg-compromising immunological tests including ELISA, dipstick, and ICT and carried out multicenter screening of the dipstick test using sera of naturally infected dogs from Brazil and Italy.

## Materials and Methods

### Study Design

The study was initiated with 80 canine sera, including 40 CVL-positive and 40 healthy dog samples collected from the Universidade Federal do Piaui, Teresina (UFPI), Brazil. Subsequently, an ELISA was performed at the Indian Institute of Chemical Biology (IICB), India, to evaluate the adequacy of LAg for *L. infantum* CVL diagnosis. Furthermore, using this antigen, the dipstick test and ICT were developed, and the tests were validated in India with Brazilian dog sera. The dipstick test was also carried out in three other CVL endemic regions, Universidade Federal da Grande Dourados (UFGD), Brazil, and Universidade Federal do Rio de Janeiro (UFRJ) with serum samples from dogs from Paraiba State, Brazil, and the University of Naples, Italy.

In UFGD, Brazil, 80 canine samples were used from two endemic municipalities in Mato Grosso do Sul state (Campo Grande and Camapuã). Fifteen samples were obtained from CVL-positive dogs for each of symptomatic, oligosymptomatic, and asymptomatic positive dogs that were diagnosed by parasitological examination of lymph node aspirates. Inaddition, 15 samples were analyzed from CVL-negative dogs (parasitological examination negative and negative serological test—DPP^®^), which were also negative for hemoparasites (polymerase chain reaction screening for *Anaplasma platys, Babesia canis*, and *Ehrlichia canis*). Yet, 15 CVL-negative naturally infected dogs were analyzed with *E. canis*, as well as five healthy vaccinated dogs (Leish-Tec^®^; Hertape Animal Health S/A, Brazil).

In UFRJ, Brazil, 125 dog sera were tested, of which 15 were *L. infantum* positives, confirmed by more than one immunological test; 56 were CVL negatives, healthy asymptomatic dogs; and 41 were *Tripanosoma cruzi* PCR–positive dogs. Thirteen *L. infantum* positive samples that were co-infected with *T. cruzi* were also included. At the University of Naples, Italy, 201 dog sera were used, which included 68 active CVL cases, 33 cured CVL, 72 healthy dogs from endemic regions of Italy, and 28 healthy dogs from non-endemic regions of Italy.

### ELISA

Leishmanial membrane antigen (LAg) was isolated from the promastigotes of *L. donovani* strain AG83 (ATCC^®^ PRA-413™) according to the protocol discussed earlier (Ejazi et al., 2020). In an indirect ELISA, Brazilian sera from UFPI were tested for antibodies against LAg. Assay conditions were determined for appropriate antigen concentration and serum dilution. Briefly, 96-well flat-bottom plates (Thermo Fisher Scientific, USA) were coated with 1.5 μg per well of LAg in coating buffer (20 mM phosphate buffer, pH 7.4) and allowed to sensitize at 4°C overnight. The next day, the remaining binding sites were blocked with 1% BSA (bovine serum albumin) in PBS (phosphate-buffered saline) and 0.05% Tween-20 for 2 h at 37°C. Afterward, the wells were incubated with dog sera at 1:2,000 dilutions, followed by 1:2,000 diluted peroxidase-conjugated anti-dog IgG, both at 37°C for 1 h. Plates were washed in each step of the reaction and finally incubated for 15 min in a substrate solution comprised of o-phenylenediamine dihydrochloride and H_2_O_2_ in the phosphate-citrate buffer at room temperature in the dark. The reaction was stopped with 2N H_2_SO_4_, and the readings were taken for optical density at 492 nm. The sensitivity of the ELISA with LAg was calculated using the receiver operating characteristic (ROC) curve.

### Dipstick Preparation and Assay

A nitrocellulose membrane–based dipstick test was developed according to the protocol reported by us earlier (Ejazi et al., 2016). Concisely, 1.5 μg of antigen LAg and 1:20 diluted anti-dog antibodies were coated for the test and control line, respectively. Subsequently, membrane blocking was done with 2% BSA and 0.1% Tween-20 plus 0.01% NaN_3_ for 8 h at 4°C. Later, the membrane was dried at 37°C for 30 min and pasted on the plastic backing. The ready-to-use dipstick was stored at room temperature under desiccation. To conduct the dipstick assay for CVL diagnosis, canine sera at 1:2,000 dilutions were incubated with the dipstick for 30 min. Subsequently, after washing, dipsticks were incubated with HRP-conjugated anti-dog IgG at a 1:2,000 dilution for 30 min, followed by washing repeats. Color development after the enzymatic reaction was done with diaminobenzidine tetrahydrochloride (Sigma, USA) supplemented with 0.05% of H_2_O_2_ in TBS (tris-buffered saline). The result was interpreted as CVL positives with two dark brown lines at the test and control positions, whereas color-only at the control line indicates a negative result.

### Multicenter Dipstick Assay

Dipsticks were manufactured at IICB, India, and transported at room temperature to Brazil and Italy for validation after quality control of the batch with confirmed Brazilian CVL-positive and CVL-negative sera. To maintain uniformity, a written protocol and a video of the dipstick assay were shared with the centers. Each center assembled the dipstick performance using CVL confirmed and pathologically classified dog sera. After conducting the test at the centers, results were shared and compiled to report the sensitivity and specificity of the test in each center.

### ICT Development and Assay

Preparation of ICT includes coating of LAg (1.5 mg/ml) and anti-dog antibody (1 mg/ml) for the test and control lines of nitrocellulose membrane (mdi Membrane Technologies, India) through a reagent dispenser (Precore Solutions, Kochi, India). Drying of the membrane took place at a 37°C incubator for 30 min. The conjugate membrane was prepared by coating colloidal-gold conjugated protein G (Ubio Biotechnology Systems, Cochin, India) with 10% sucrose in PBS buffer and drying at a 37°C incubator for 30 min. The sample membrane, conjugate membrane, nitrocellulose membrane, and absorbent membrane were assembled together and cut into 2.5-mm strips to place in a diagnostic cassette until use. For the assay, 10 μl of dog serum was applied at the sample membrane position, followed by two drops of chase buffer containing 20 mM tris (pH 7.4 with 0.1% Tween-20). After 5 min, color bands in the nitrocellulose membranes were visualized in which color-only on the control line showed a CVL-negative samples, whereas colors in both the test and control lines depicted CVL-positive sera.

### Statistical Analysis

For statistical analysis, GraphPad Prism version 5 was used. Values between positive and negative groups were compared with the Mann–Whitney U-test and judged statistically significant if the P-values were less than 0.05. The cutoff value for ELISA was determined using an ROC curve with 95% confidence intervals (CI). Sensitivity and specificity were calculated to measure the test’s overall performance, and diagnostic accuracy was determined using the area under the curve (AUC), with AUC = 1 indicating an accurate test.

## Results

### LAg ELISA for the Diagnosis of CVL

**Figure 1:**
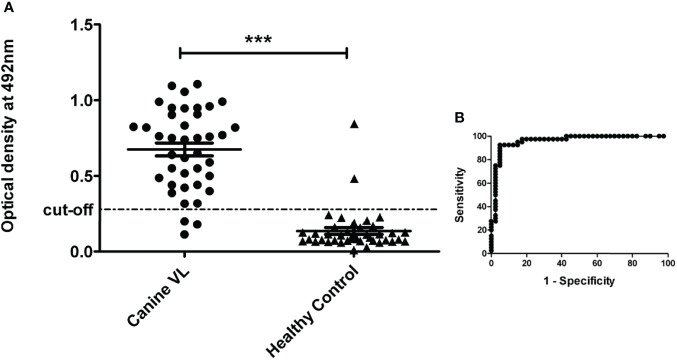
Indirect ELISA to detect antibodies in canine sera against leishmanial antigen LAg. **(A)** Brazilian CVL samples (n = 40) and healthy dogs sera (n = 40) were used in this study. Each dot represents an average value of a single sample. The significance between the groups was obtained as *p* < 0.0001 (***). **(B)** Cutoff was selected according to the receiver operator characteristic (ROC) curve obtained from GraphPad Prism 5.

The serological analysis of 80 Brazilian dogs based on the LAg antigen in ELISA was conducted in India. The sensitivity was calculated using the generated cutoff through the ROC curve (**Figure 1**). A total of 37 of the 40 CVL samples showed the antibody titer above the cutoff, hence illustrating positives for the test with 92.50% (95% CI: 79.61% to 98.43%) sensitivity (**Table 1**). The antibody titer in three CVL samples was not enough to accomplish the cutoff; therefore, they were considered false negatives. The assay was also evaluated with the sera of 40 healthy endemic dogs as a control. At the set cutoff, 38 samples were found to be negative for the ELISA, contributing to a 95% (95% CI: 83.08% to 99.39%) specificity. Two healthy dog sera were cross-reactive to the antigen as their titer was above the cutoff. The area under the ROC curve was 0.96 for the ELISA.

**Table 1:** Performance of LAg-based ELISA, dipstick test, and ICT with sera from Universidade Federal do Piaui (UFPI), Brazil.

	Cases	No. of Samples	LAg Positives	LAg Negatives	Sensitivity	Specificity
ELISA	CVL	40	37	3	92.50%	–
	Healthy endemic canine	40	2	38	–	95%
Dipstick	CVL	39	36	3	92.30%	–
	Healthy endemic canine	39	2	37	–	94.87%
ICT	CVL	38	36	2	94.73%	–
	Healthy endemic canine	37	1	36	–	97.29%

### Dipstick Test Development and Multicenter Evaluation

**Figure 2:**
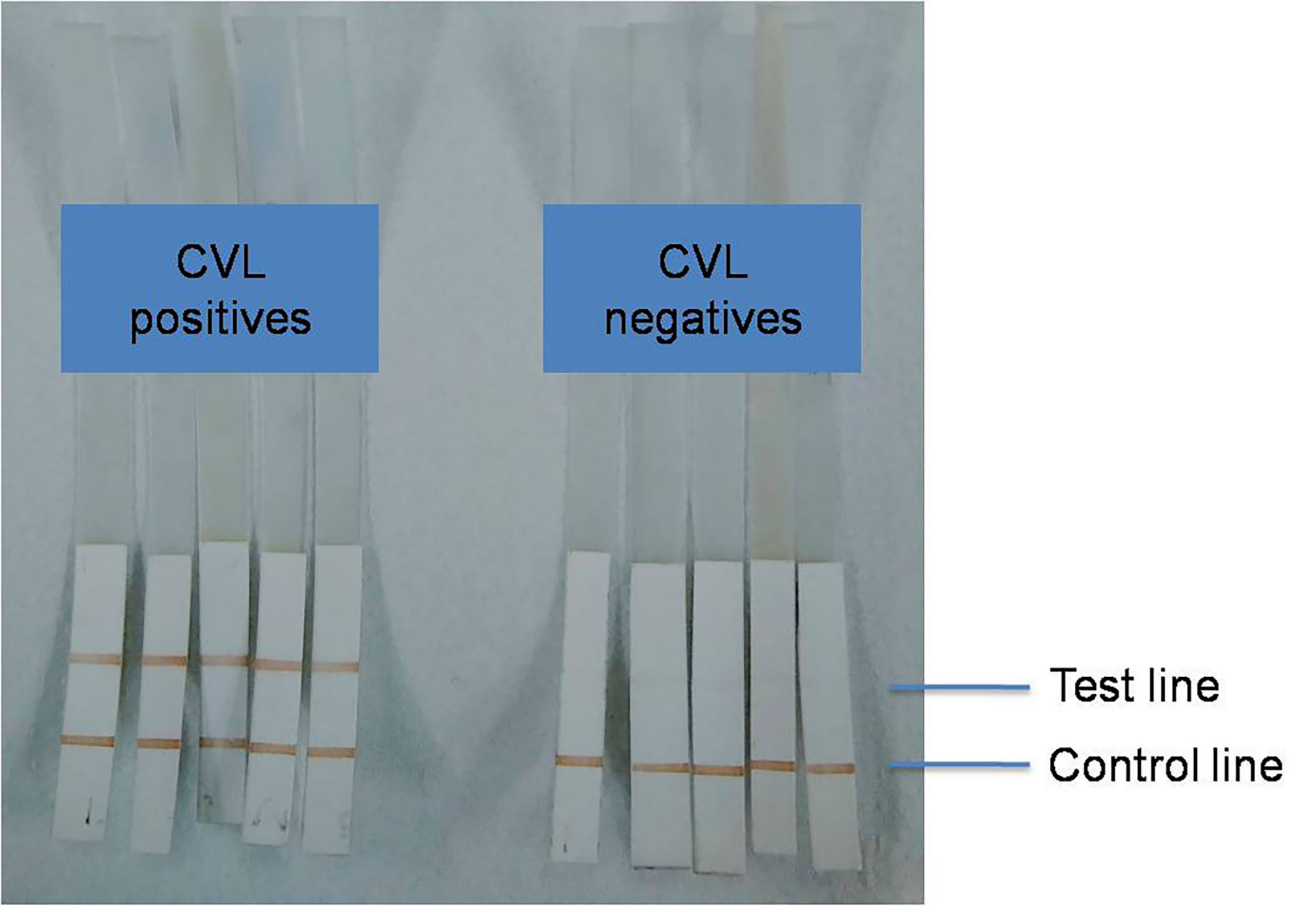
Representative result of dipstick test with positive and negative CVL sera.

Following the evaluation of ELISA, a nitrocellulose membrane–based dipstick test was developed for CVL diagnosis using antigen LAg. This LAg-coated dipstick was designed for serological detection of CVL in a comparatively simple and less time-consuming manner than ELISA. The color bands obtained at the test line were due to the enzymatic reaction promoted by the reactivity of LAg with the antibodies present in infected canine sera (**Figure 2**). The dipsticks were initially validated in India with Brazilian serum samples collected from the UFPI and were later transported to UFGD and UFRJ, both in Brazil, as well as the University of Naples in Italy, for multicenter validation.

#### India

A total of 78 canine sera from UFPI, Brazil, were tested in India, including 39 samples from parasitologically confirmed CVL cases and 39 samples from healthy dogs from the region. Analysis of the positive canine sera from Piaui, Brazil, through the dipstick test showed 36 of 39 positives, resulting in 92.30% sensitivity to diagnose CVL (**Table 1**). Three of the 39 CVL samples were missed by dipsticks. Of 39 healthy dogs’ samples, 37 sera were negative in the dipstick assay. As a result, the test’s specificity for distinguishing healthy dogs from infected dogs was found to be 94.87%, despite the fact that two healthy dog sera were cross-reactive for the test.

#### Brazil

A blind fold study was conducted at UFGD, for the evaluation of LAg-based dipstick in CVL diagnosis. All the 80 canine samples used for the assay were pathologically characterized samples that were tested by dipsticks and also compared with the DPP^®^ test. Reactivity of LAg-dipsticks with sera collected from symptomatic and oligosymptomatic dogs showed 73.33% and 86.66% sensitivity, respectively, as compared to DPP^®^, which had a sensitivity of 100% with both dog groups (**Table 2**). For detecting asymptomatic CVL, dipsticks recognized 46.66% of dogs, whereas 53.33% of asymptomatic dogs were recognized by the DPP^®^ test. Similar to DPP^®^, the dipstick test demonstrated 100% specificity with CVL-negative dogs and dogs infected with *E. canis*. Five healthy vaccinated dogs were also found negative in dipstick tests.

**Table 2:** Performance of dipstick test at Universidade Federal da Grande Dourados (UFGD), Brazil.

Cases	No. of Samples	DPP^®^ Rapid Test		Dipstick Test		
		Positive	Negative	Positive	Negative	
Symptomatic	15	15	0	11	4	Sensitivity 73.33%
Oligo-symptomatic	15	15	0	13	1	Sensitivity 86.66% (1 invalid)
Asymptomatic	15	8	7	7	8	Sensitivity 46.66%
Control CVL-negative and hemoparasites negative	15	0	15	0	15	Specificity 100%
Control with *Ehrlichia canis*-CVL negative	15	0	15	0	15	Specificity 100%
Vaccinated	5	0	5	0	5	Specificity 100%

At the center of UFRJ, 125 samples were tested by the dipstick assay. Fifteen samples were evaluated by the dipsticks that were tested as CVL positive for more than one immunological test (DPP^®^, ELISA Biomanguinhos^®^, and ELISA S7^®^Biogene^®^). Eight samples were found positive in this group in the dipstick assay, showing 53.33% sensitivity (**Table 3**). Moreover, four samples were recognized by the dipstick test out of 13 *L. infantum* and *T. cruzi* co-infected dogs (tested with Nested PCR), indicating 30.76% reactivity. The dipstick test with 56 healthy asymptomatic and 41 *T. cruzi* positive dogs yielded 98.21% and 97.56% specificity, respectively, resulting from cross-reactivity with one sample each.

**Table 3:** Performance of dipstick test at Universidade Federal do Rio de Janeiro (UFRJ) on pre-diagnosed dogs from endemic Paraiba State, Brazil.

Cases	No. of Samples	Dipstick Positives	Dipstick Negatives	Sensitivity	Specificity
Confirmed *L. infantum* positive canine sera	15	8	7	53.33%	–
*L. infantum* plus *T. cruzi* co-infection positive canine sera	13	4	9	30.76%	–
Healthy asymptomatic dogs	56	1	55	–	98.21%
*T. cruzi* positive dogs	41	1	40	–	97.56%

#### Italy

Dipsticks were evaluated at the University of Naples using 201 serum samples collected from different groups of dogs. Sensitivity of 100% was achieved in dipstick assay using 68 molecular (qPCR) method confirmed CVL cases without any false negatives. Of 33 cured CVL, except for one case, all dogs’ sera showed positivity with the dipsticks, resulting in 96.96% sensitivity (**Table 4**). The dipstick, test with 28 healthy dogs from non-endemic regions was found to be 100% specific. However, 72 sera collected from healthy dogs in endemic regions showed 34.73% cross-reactivity.

**Table 4:** Performance of dipstick test at University of Naples, Italy.

Cases	No. of Samples	Dipstick Positives	Dipstick Negatives	Sensitivity	Specificity
Confirmed CVL	68	68	0	100%	–
Cured CVL	33	32	1	96.96%	–
Healthy endemic canine	72	25	47	–	65.27%
Healthy nonendemic canine	28	0	28	–	100%

### Colloidal-Gold Based ICT for CVL Diagnosis

**Figure 3:**
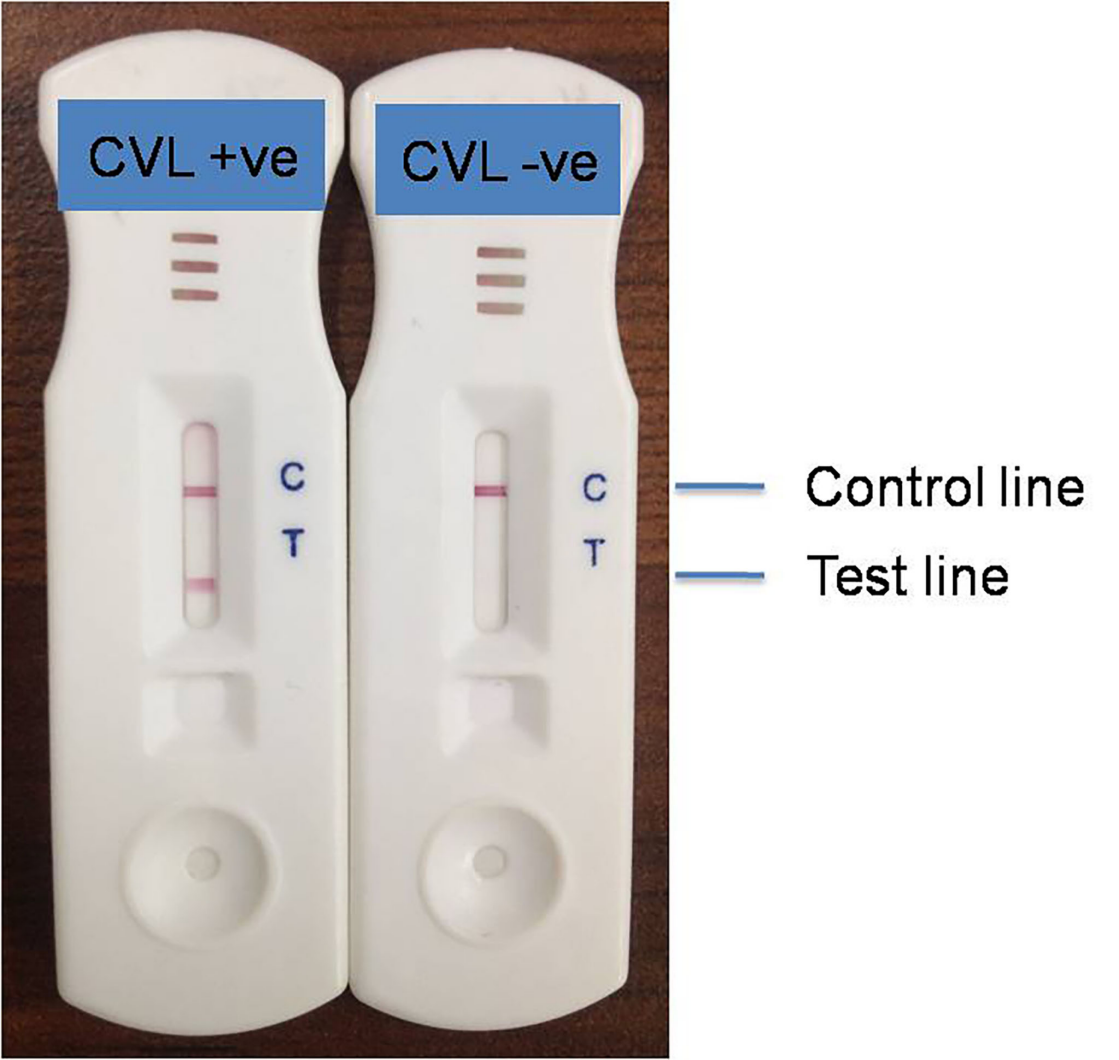
Representative result of ICT with positive and negative CVL sera.

A colloidal gold–based ICT test was developed to investigate the possibility of leishmanial antigen, LAg, for CVL diagnosis in field settings. Depending upon the infection, *Leishmania*-specific antibodies bind to the coated antigen at the test line. The anti-canine antibody at the control line binds with the sample antibodies as an experimental control. Positive reactivity at the test and control lines can be seen visually due to the gold conjugates (**Figure 3**). After optimization of the LAg-based ICT for CVL diagnosis, 75 Brazilian dog sera from UFPI were tested to determine the sensitivity and specificity of the newly developed ICT. The ICT had a sensitivity value of 94.73% for a total of 38 confirmed CVL cases (**Table 1**). Two positive samples did not give color at the test line as false negatives. In 37 healthy dog sera, ICT distinguished 36 sera as negatives, and a false positive reactivity concluded with 97.29% specificity.

## Discussion

Infection due to *L. infantum* is mainly zoonotic where dogs as parasite reservoirs are associated with a risk of disease transmission to humans. To reduce the transmission through a vector, early diagnosis of the infected animals is highly desirable. Therefore, one of the mainstays of the VL elimination program in the endemic zoonotic areas is proper CVL case detection. In this study, we have developed and tested immunological assays such as ELISA, dipstick, and ICT for the serological diagnosis of CVL against the LAg and validated its performance in several CVL endemic regions of Brazil and Italy, demonstrating strong and specific recognition of canine IgG anti–*L. infantum* antibodies by the LAg.

The presence of a large number of subclinical or asymptomatic infections and variations in clinical signs is the major obstacle to single out the infected dogs. A conventional method of detecting amastigotes following biopsy or microscopy is invasive and time-consuming and has limited throughput for large-scale application. A variety of immunological techniques have been employed in CVL diagnosis that is related to the detection of *Leishmania*-specific antibodies in infected dogs. However, the presence of a large amount of antibodies as immune complexes may lead to false negative results in serology (Aparecida de Carvalho et al., 2021). Therefore, the success of immunological diagnosis in terms of sensitivity and specificity mainly depends on the ability of the antigens used in the assay. So far, there is no single antigen that can be facilitated in the acquisition of screening CVL. Because of the low sensitivity of recombinant antigens, the use of crude antigens or cocktails and the fusion of more than one antigen are still in practice for CVL diagnosis. The current recommendation for detecting CVL in Brazil is to first screen the sample with a DPP^®^ CVL test based on rK28 antigen followed by confirmation through crude antigen–based ELISA (Souza et al., 2019). A recent review found that soluble antigen, or SLA, was employed in 53.8% of publications on antigen-based CVL diagnosis between 2016 and 2021 (Barbosa de Castro et al., 2022). In a study, *L. infantum* crude antigen in an agglutination test showed 100% sensitivity for symptomatic VL dogs as compared to 32.4% and 52.9% with rKE16 and rA2 (Farahmand et al., 2015). Recently, antibody response in experimental canine leishmaniasis has suggested the utility of only promastigote antigen in early CVL diagnosis (Olías-Molero et al., 2019).

We have earlier reported the diagnostic ability of LAg against both *L. donovani–* and *L. infantum–*infected human VL (Ejazi et al., 2019; Ejazi et al., 2021). In a dipstick format, LAg has been validated in eight centers in six countries, including India, Nepal, Sri Lanka, Brazil, Ethiopia, and Spain, and found overall 97.10% sensitivity and 93.44% specificity (Ejazi et al., 2019). In this context, LAg was tested in the current work for CVL diagnosis in several endemic regions. Examining Brazilian canine sera in LAg-ELISA showed 92.50% sensitivity and 95% specificity in diagnosing CVL. In comparison with a similar study in Brazil, crude antigen has demonstrated 75% sensitivity and 73.3% specificity in chemiluminescent ELISA. However, with a multi-epitope protein, PQ10, the sensitivity and specificity have been reported to be 93% and 80%, respectively (Fonseca et al., 2019). Therefore, in the preliminary investigation, LAg-ELISA showed acceptable sensitivity and specificity in identifying canine VL.

To overcome the limitations of ELISA, most notably the long assay time and the need for sophisticated equipment, the dipstick test was developed, which is simpler and faster than ELISA. The LAg-based dipstick test with Brazilian canine samples from UFPI showed 92.30% sensitivity for CVL confirmed sera and 94.87% specificity to distinguish healthy dog sera from CVL infection. The result is in concordance with our earlier study, where we found 100% sensitivity and specificity of LAg-dipstick in diagnosing *L. infantum*–infected human VL in Brazil (Ejazi et al., 2019). The fact that the performance of antigens is affected across different geographical regions led to the validation of diagnostic tests in distinct locations as part of a multicenter study. In this present study, we have evaluated the dipstick test in the endemic areas of Brazil and Italy. The LAg-dipstick at the UFGD, Brazil, has been found to be 73.33%, 86.66%, and 46.66% sensitive in detecting symptomatic, oligosymptomatic, and asymptomatic CVL dogs, respectively. The dipstick test in this region showed 100% specificity with CVL-negative dogs and *E. canis–*infected dogs, similar to the commercially available DPP^®^ test. Importantly, in this study, the dipstick test did not react against sera from dogs that were pre-vaccinated with the commercial LeishTec^®^ anti-CVL vaccine, unlike many tests where antibodies generated in response to the vaccine cross-reacted with the antigens. The dipstick test assayed in another Brazilian center of UFRJ using dog samples collected from another endemic area of Brazil, the Paraiba State, showed a sensitivity of 53.33% for confirmed CVL cases. In the case of human VL, several reports have established that the performance of serological tests, although excellent in VL diagnosis has discovered a significant decrease in its sensitivity against VL/HIV (Cota et al., 2012). Therefore, we sought to observe the difference in the performance of our dipstick test with or without coinfection. As can be seen, the dipstick sensitivity reduced to 30.76% with *Trypanosoma* coinfection from 53.33% for only *Leishmania* infection. Therefore, we observe a variance in dipstick sensitivity due to the coinfection. The specificity of the dipstick test with control sera, including healthy asymptomatic and *T. cruzi* positive samples, was found to be 98.21% and 97.56%, respectively. Several newer endemic foci of CVL have been recently discovered in Italy (Gradoni et al., 2022). We validated our dipsticks in a center of the University of Naples, Italy. The dipstick assay achieved 100% sensitivity and specificity in CVL diagnosis as compared to healthy non-endemic dogs. However, the dipsticks still recognized 96.96% of cured CVL sera as well as 34.73% of healthy endemic dogs. The overall performance of the dipstick test in four centers was found to be 89.78% sensitive for symptomatic CVL cases and 86.66% specific with respect to endemic and non-endemic healthy dogs. The inconsistency that we record in the performance of dipstick at different centers can be due to the distinct disease prevalence across the region as many recent studies support this. A study in three different Brazilian states has shown sensitivities ranging from 50% to 94.23% against the soluble leishmanial antigen (Ramirez et al., 2019). The geographical location of the infected dogs influenced serological detection. In addition, the diagnostic efficacy was found to be higher in newly endemic regions than in areas of more endemicity.

As the results found in the dipstick assay were comparable and highly satisfactory, LAg was further used to develop a point-of-care test for CVL diagnosis in the form of ICT. Lateral flow–based ICT has the advantage of being completed in a few minutes with simplicity and can be interpreted directly as visible bands. In recent years, several antigens have been reported in ICT format. In one study, three recombinant antigens, rFc, rC9, and rA2, were tested against CVL and found to be 88.6%, 86.5%, and 87% sensitive, respectively (Santos et al., 2019). Identification of several newer antigens of *L. infantum* in Brazil demonstrated sensitivities between 49% and 97% for CVL diagnosis in comparison with 93% and 68% sensitivity with parasite lysate and rK39 antigen, respectively (Magalhaes et al., 2017). Earlier, LAg in ICT format had been evaluated with Brazilian human sera and had 88.57% and 94.73% sensitivity and specificity, respectively (Ejazi et al., 2021). In this study, we have accessed the performance of LAg-ICT against Brazilian canine sera. The results acquired from the LAg-ICT have identified 94.73% of confirmed CVL cases, with a specificity of 97.29%. This result indicated that the developed ICT could be used to diagnose canine VL through a rapid ICT test.

In conclusion, LAg, as used in this study in ELISA, dipstick, and ICT assays, demonstrated high reactivity with antibodies present in *L. infantum–*infected dogs. Thus, this antigen could be employed for serodiagnosis of CVL in zoonotic regions. Nevertheless, the performance of LAg in diagnosing CVL in the current study is found to be as good as its performance in detecting human VL in our previous studies. However, the study warrants further validation of the test with more defined groups of CVL in the future.

## Data Availability Statement

The original contributions presented in the study are included in the article/supplementary material. Further inquiries can be directed to the corresponding author.

## Ethics Statement

The animal study was reviewed and approved by Committee on the Ethics of Animal Use (CEUA) of the Universidade Federal de Campina Grande, 059/2018 and 52/2018; Internal review Board, CSIR-IICB/24.01.2017; Ethical Committee from the Universidade Federal do Piauí #116/2005; and Universidade Federal da Grande Dourados, 645/2014 and 697/2015.

## Author Contributions

SE and SS planned and executed the study, developed and performed assays, evaluated results, interpreted the data, and wrote the manuscript. AB and SD performed assays and evaluated the results. NM, MJ, and HN-A performed dipstick test at UFGD, Brazil. IM and CC provided dogs sera and performed sera profiling at UFPI, Brazil. OM, MM, and BR-B performed dipstick test at UFRJ, Brazil. RC and LM performed dipstick test at University Federico II of Naples, Italy. NA, conceptualized the study,data interpreted and wrote the manuscript. All authors contributed to the article and approved the submitted version.

## Funding

This work has, in part, received funding from UK Research and Innovation *via* the Global Challenges Research Fund under grant agreement “A Global Network for Neglected Tropical Diseases” grant number MR/P027989/1; Sir J. C. Bose Fellowship, India; and Council of Scientific and Industrial Research, India.

## Conflict of Interest

The authors declare that the research was conducted in the absence of any commercial or financial relationships that could be construed as a potential conflict of interest.

## Publisher’s Note

All claims expressed in this article are solely those of the authors and do not necessarily represent those of their affiliated organizations, or those of the publisher, the editors and the reviewers. Any product that may be evaluated in this article, or claim that may be made by its manufacturer, is not guaranteed or endorsed by the publisher.
